# Odium Medicum and Homœopathy

**Published:** 1888-06

**Authors:** 


ODIUM MEDICUM AND HOMŒOPATHY.

This pamphlet, of one hundred and twenty-six pages, is a reprint, with additions, from the London *Times* of a spicy correspondence upon Homœopathy. It is edited by Dr. John H. Clarke, of London, and is very well worth a perusal. The allopathic defamers of Homœopathy offer nothing new in the way of argument or proof against it. Merely dealing, as usual, in assertion, ridicule, and bombast, they retired from the discussion, gaining little beyond the contempt of all fair-minded persons.

